# Retrospective cohort analysis on predicting pulmonary fibrosis in elderly SARS-CoV-2-infected patients

**DOI:** 10.3389/fcimb.2025.1587321

**Published:** 2025-06-06

**Authors:** Fuguo Gao, Guangdong Hou, Yan Hou, Jian Chen, Yifeng Wang, Baoyin Zhao, Yan Li, Xinxin Wang, Yiying Hua, Faguang Jin, Yongheng Gao

**Affiliations:** ^1^ Department of Pulmonary and Critical Care Medicine, Tangdu Hospital, The Fourth Military Medical University, Xi’an, China; ^2^ Department of Pulmonary and Critical Care Medicine, The 940th Hospital of the Joint Logistics Support Force of People’s Liberation Army (PLA), Lanzhou, China; ^3^ Department of Urology, Tangdu Hospital, The Fourth Military Medical University, Xi’an, China; ^4^ Department of Pulmonary and Critical Care Medicine, Shaanxi Provincial People’s Hospital, Xi’an, China

**Keywords:** elderly, SARS-CoV-2, pulmonary fibrosis, prediction model, neutrophil percentage

## Abstract

**Background:**

SARS-CoV-2 exhibits rapid transmission with a high susceptibility rate, particularly among the elderly. Pulmonary fibrosis (PF) following SARS-CoV-2 infection is a life-threatening complication. However, predictive models for PF in older patients are lacking.

**Methods:**

Data from patients with COVID-19 aged 60 and above, collected retrospectively between November 2022 and November 2023 across two independent hospitals, were analyzed. Patients from Tangdu Hospital were divided into training and validation cohorts using a 7:3 allocation ratio, while those from The 940th Hospital of the Joint Logistics Support Force of the People’s Liberation Army (PLA) served as the test cohort. Identify the most valuable predictors (MVPs) for PF using Least Absolute Shrinkage and Selection Operator (LASSO) regression, and construct a nomogram based on their regression coefficients derived from logistic regression. The calibration, clinical utility, and discriminatory ability of the nomogram were evaluated using the Hosmer-Lemeshow test, decision curve analysis (DCA), and Receiver Operating Characteristic (ROC) curve, respectively.

**Results:**

Neutrophil percentage, C-reactive protein (CRP), gender, diagnostic classification, and time from symptom onset to hospitalization were identified as the MVPs for PF. The nomogram was developed based on these predictors, In all the three cohorts, the nomogram showed good calibration, clinical utility and discriminatory ability, with Area Under the Curve (AUC) of 0.777, 0.735 and 0.753, respectively. Furthermore, based on the principle of optimizing the balance between sensitivity and specificity, 131.026 was determined as the optimal cutoff value for the nomogram. Accordingly, patients with a nomogram score of 131.026 or higher were classified into the high-risk group.

**Conclusions:**

This study presents the first nomogram for predicting PF in elderly patients following SARS-CoV-2 infection, which may serve as a clinical tool for risk assessment and early management in this population.

## Introduction

SARS-CoV-2 exhibits rapid spread and extensive transmission, with heightened susceptibility within populations compared to other influenza viruses. Symptoms following infection are more pronounced (Pavia et al., 2024). Among those infected, a significant proportion will demonstrate lung imaging changes and may develop pulmonary fibrosis (PF) (Cui et al., 2023). Current clinical diagnosis of PF relies on a comprehensive evaluation involving high-resolution computed tomography (HRCT) and pulmonary function tests, though their sensitivity is limited in the early stages of the disease. During the initial infectious phase, prior to fibrotic formation, HRCT typically reveals non-specific alterations, such as ground-glass opacities (Sverzellati et al., 2021). Patients with subclinical or mild symptoms are often less inclined to undergo systematic imaging, complicating early detection. Pulmonary function tests, including forced vital capacity (FVC) and diffusing capacity for carbon monoxide (DLCO), also exhibit insufficient sensitivity and specificity in the early stages (Guiot et al., 2024), especially when no significant fibrotic changes in lung architecture are observed. This diagnostic uncertainty complicates differentiation between viral pneumonia and the progression of interstitial lung disease in the early pathological phase.

PF is a life-threatening condition that severely impacts patients’ quality of life. Treatment options remain limited, with early diagnosis and intervention being the primary strategies to reduce the clinical burden of PF (Li et al., 2023). Recent research has highlighted a close association between age and the development of PF following SARS-CoV-2 infection (Ribeiro Carvalho et al., 2024), with advanced age identified as a risk factor influencing disease severity and prognosis (Cui et al., 2022). Furthermore, many elderly patients do not present typical symptoms such as fever or respiratory distress in the early stages of the disease (Graham et al., 2020), resulting in delayed diagnosis and missed opportunities for early intervention. Early identification and targeted management of elderly patients with SARS-CoV-2 infection, particularly those at high risk of developing PF, are critical to preventing PF onset and reducing associated mortality. Current prediction models for assessing post-SARS-CoV-2 infection risks primarily focus on the risk of death following severe infection and the survival rates of patients with SARS-CoV-2 pneumonia (Dong et al., 2021; Yang et al., 2021). However, prediction models specifically targeting PF progression in the elderly after SARS-CoV-2 infection remain lacking, resulting in delayed identification of PF in these patients and impacting their survival prognosis (Hirawat et al., 2023). This underscores the urgent need for early warning research focused on PF development in the elderly and the creation of prediction models that integrate relevant variables to assess the risk of progression to PF after SARS-CoV-2 infection.

Consequently, this study explored the risk factors associated with PF progression in elderly SARS-CoV-2-infected patients and developed a high-performance prognostic model that integrates multidimensional predictors, including clinical characteristics, laboratory parameters, and temporal factors. The resulting early warning system facilitates the timely prediction of PF outcomes in elderly patients with COVID-19, playing a pivotal role in optimizing clinical management, improving prognosis, and enhancing quality of life.

## Methods

### Study design and population

This study retrospectively analyzed patients aged 60 years or older who were infected with the novel coronavirus and treated at Tangdu Hospital of The Fourth Military Medical University and The 940th Hospital of the Joint Logistics Support Force of the People’s Liberation Army (PLA) between November 2022 and November 2023. Inclusion criteria were: (1) A diagnosis of novel coronavirus infection according to the “Diagnosis and Treatment Protocol for SARS-CoV-2 Virus Infection (Trial Version 9)”; (2) Being aged 60 years or older; (3) The availability of complete imaging and laboratory test results. Exclusion criteria included: (1) Having a history of idiopathic or secondary PF; (2) Currently using drugs that may induce PF (e.g., amiodarone) or treatments that may induce PF (e.g., radiotherapy); (3) Missing clinical or imaging data; (4) A clinical diagnosis of critical illness. A total of 661 patients from Tangdu Hospital were randomly divided into training and validation cohorts in a 7:3 ratio, while 196 patients from the 940th Hospital served as the test cohort. Data entry was performed by two individuals and verified for accuracy. Any missing or erroneous data were promptly identified and corrected. Chest HRCT scans were assessed by two senior clinical doctors specializing in respiratory medicine, with disagreements resolved through discussions led by the chief physician of chest imaging until consensus was reached.

### Research procedures

This study collected a range of potential predictive factors, including age, gender, height, weight, surgical history, blood transfusion history, past medical history (e.g., chronic obstructive pulmonary disease [COPD], asthma, pulmonary tuberculosis, diabetes, hypertension, coronary heart disease, autoimmune diseases), C-reactive protein (CRP), D-dimer, white blood cell count, lymphocyte count, neutrophil count, eosinophil count, percentage of neutrophils, percentage of lymphocytes, and the time from symptom onset to hospitalization. The time from symptom onset to hospitalization was categorized into three segments: TIME 1 ≤ 1 week, 1 week < TIME 2 ≤ 2 weeks, and 2 weeks < TIME 3.

### Statistical analysis

The Kolmogorov-Smirnov test was utilized to assess the distribution characteristics of the data. For normally distributed continuous variables, descriptive statistics were expressed as mean *±* standard deviation ( $\bar{x}$ *± s*). Group comparisons were conducted using the independent-sample t-test with a two-sided hypothesis, where a *P*-value < 0.05 was considered statistically significant. For non-normally distributed data, results were presented as median and interquartile range (IQR), and comparisons were performed using the Mann-Whitney U test. Categorical variables were reported as frequency and percentage, with the chi-square test used to compare categorical data between groups.

To reduce dimensionality and identify the most valuable predictors (MVPs), the study employed Least Absolute Shrinkage and Selection Operator (LASSO) regression analysis combined with 10-fold cross-validation. MVPs, based on regression coefficients, were integrated into a binary logistic regression model to develop a nomogram. The discriminatory ability of individual or combined variables for PF following SARS-CoV-2 infection was assessed using the receiver operating characteristic (ROC) curve. Meanwhile, the Hosmer-Lemeshow goodness-of-fit test was used to evaluate the calibration of the nomogram, accompanied by the calibration plot. The decision curve analysis (DCA) was used to determine the utility of the nomogram in clinical decision-making. Statistical significance was set at a two-sided *P*-value < 0.05. All statistical analyses were performed using the Statistical Package for the Social Sciences (SPSS) version 26.0 software and R version 4.2.3 software.

## Results

### General characteristics

**Figure 1:**
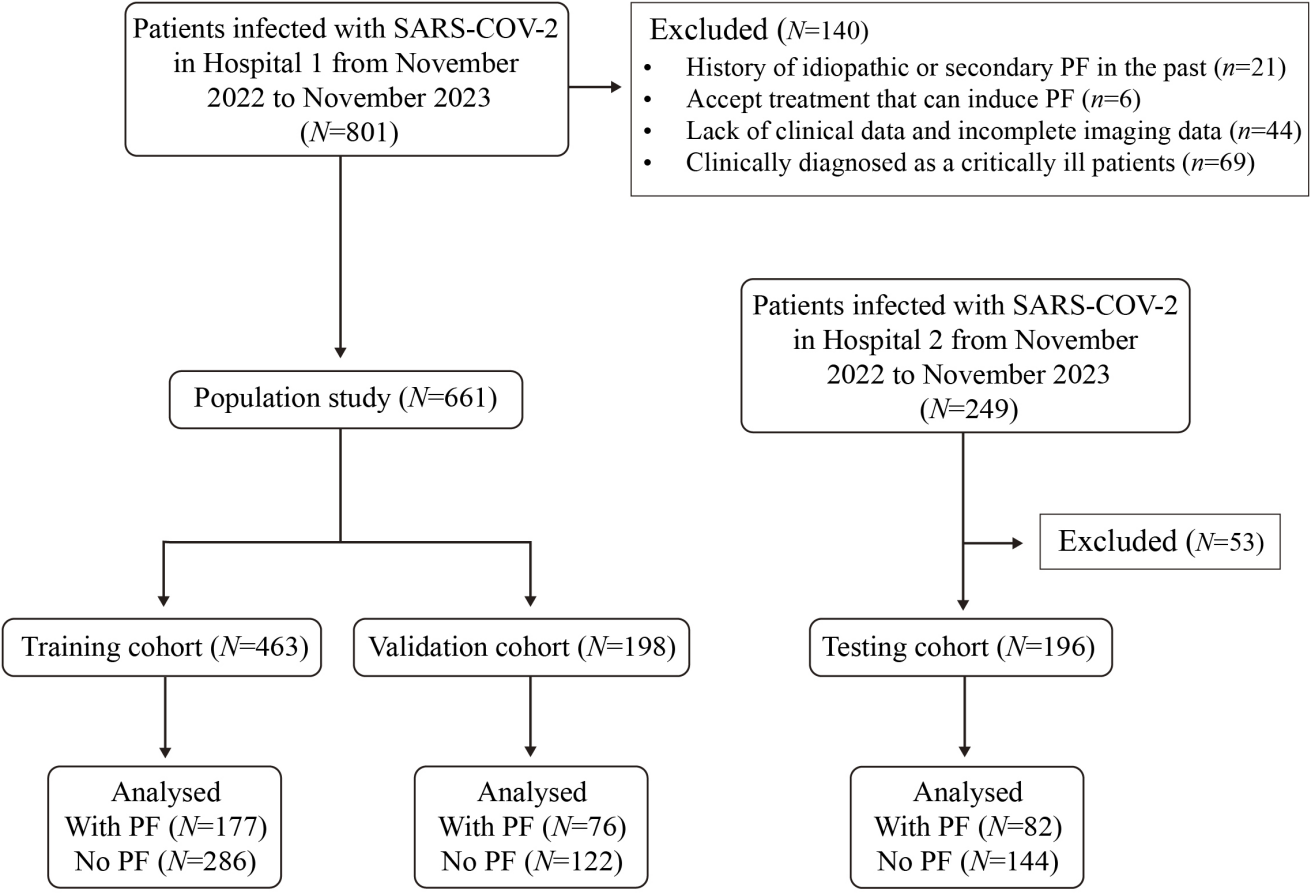
The flow chat of this study. The training and validation cohort (7:3) were collected in Hospital 1. A testing cohort for the model was collected in Hospital 2.

**Figure 2:**
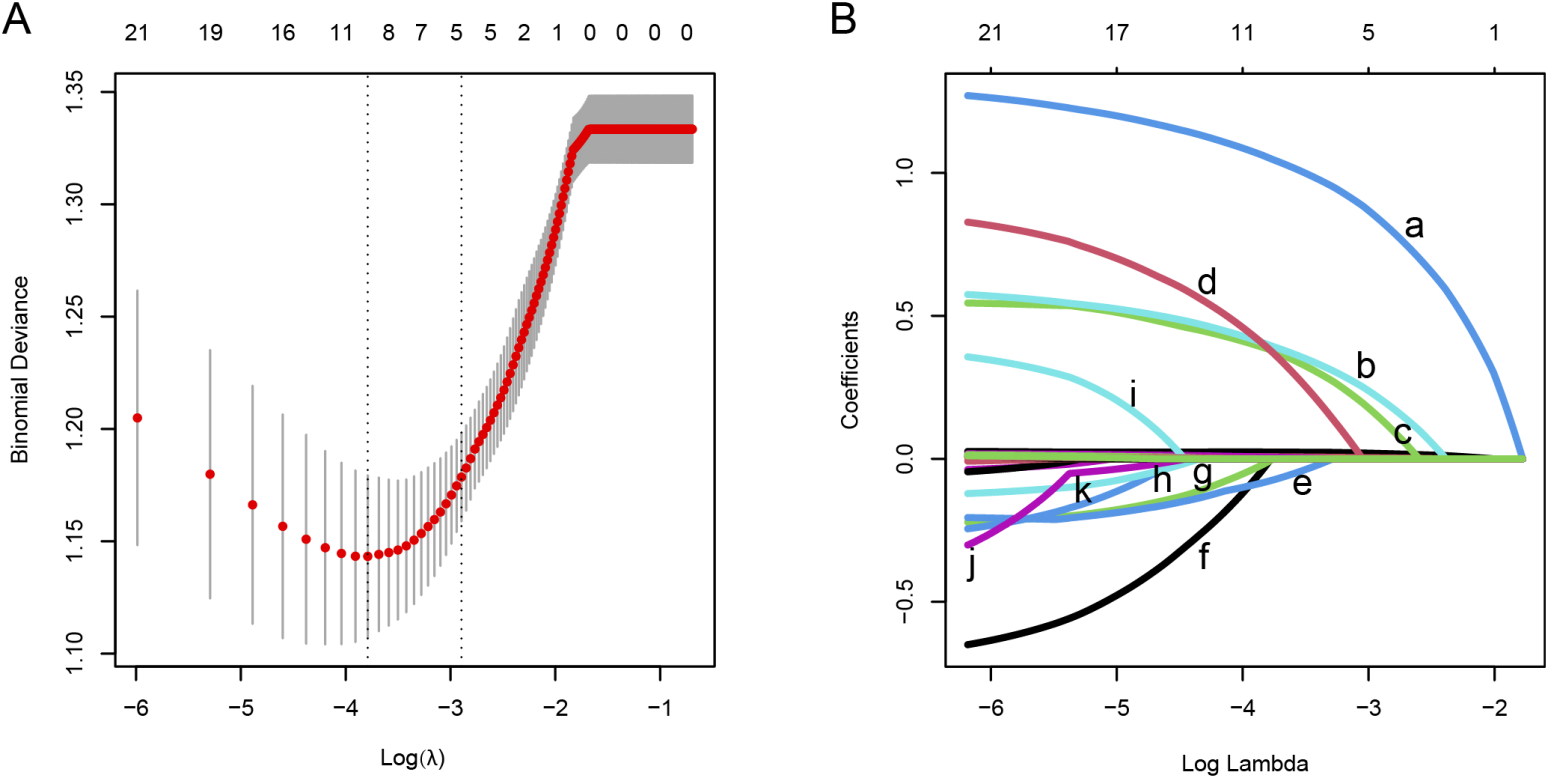
Feature screening was performed using the LASSO regression analysis combined with the 10-fold cross-validation method. **(A)** The interrelationships among the logarithm (*λ*), Mean Squared Error (MSE), and the number of variables in the model. **(B)** The LASSO coefficient curves of the candidate features. The intersecting curves indicate the number of features retained at a specific log(*λ*) value. Based on the standard error, 5 key predictors with non-zero coefficients were selected.

The research flow chart is depicted in **Figure 1**. This study included 463, 198, and 196 cases in the training, validation, and test cohorts, respectively. Among these, 177 cases (38.2%), 76 cases (38.4%), and 82 cases (41.8%) developed PF. A representative HRCT image of PF in SARS-CoV-2-infected patients is shown in **Supplementary Figure 1**. LASSO regression analysis with 10-fold cross-validation was employed to identify the MVPs for PF. Furthermore, according to the ‘one standard error’ method, 5 MVPs for PF were identified, which were the percentage of neutrophils, CRP, gender, diagnostic classification, and the time from the onset of symptoms to hospitalization (**Figure 2**).

In the training cohort, the Area Under the Curve (AUC) values of these 5 predictors and their 95% confidence intervals (CI) were 0.675 (0.625 - 0.724), 0.662 (0.612 - 0.712), 0.586 (0.533 - 0.638), 0.675 (0.623 - 0.726), and 0.611 (0.559 - 0.664) respectively. In the validation cohort, the corresponding AUC values and their (95% CI) were 0.644 (0.564 - 0.724), 0.672 (0.596 - 0.748), 0.575 (0.494 - 0.656), 0.669 (0.590 - 0.748), and 0.582 (0.500 - 0.664). In the test cohort, the AUC values and (95% CI) of the 5 predictors were 0.672 (0.596 - 0.747), 0.656 (0.577 - 0.734), 0.567 (0.486 - 0.648), 0.639 (0.560 - 0.718), and 0.627 (0.548 - 0.706).

**Table 1** presents the distribution of characteristics for both the training and validation cohorts, as well as the training and test cohorts.

**Table 1:** Characteristics analysis of research participants.

Variable	Training (*n*=463)	Validation (*n*=198)	*P*
Gender			0.533
Male	304 (65.66)	125 (63.13)	
Female	159 (34.34)	73 (36.87)	
Age	72 (67-80)	69 (66-77)	0.201
BMI	23 (21.22-25.1)	23.59 (20.76-25.95)	0.255
Operation history	224 (48.38)	92 (46.46)	0.652
BTH	29 (6.26)	14 (7.07)	0.700
COPD	35 (7.56)	7 (3.54)	0.052
Diabetes	114 (24.62)	44 (22.22)	0.508
Hypertension	221 (47.73)	91 (45.96)	0.676
CHD	103 (22.25)	31 (15.66)	0.054
AID	18 (3.89)	14 (7.07)	0.081
Cough	420 (90.71)	175 (88.38)	0.360
Fever	389 (84.02)	162 (81.82)	0.487
Diagnostic classification			0.905
Common type	283 (61.12)	122 (61.62)	
Heavy type	180 (38.88)	76 (38.38)	
Time			0.592
≤7 Days	162 (34.99)	75 (37.88)	
(7–14 ] Days	171 (36.93)	65 (32.83)	
>14 Days	130 (28.08)	58 (29.29)	
WBC (×10^9^)	6.19 (4.66-8.57)	5.99 (4.77-8.39)	0.948
NEUT (×10^9^)	4.67 (3.15-7.14)	4.46 (3.4-6.56)	0.732
LYM (×10^9^)	0.82 (0.51-1.175)	0.91 (0.63-1.23)	0.076
EOS (×10^9^)	0.02 (0-0.075)	0.04 (0-0.09)	0.071
LYMs%	13.9 (7.25-21.2)	15.9 (10.8-21.6)	0.129
NEUT%	76.3 (67.5-85.35)	74.1 (67.1-81.1)	0.087
RBC (×10^12^)	4.01 (3.53-4.38)	3.99 (3.42-4.31)	0.543
Hb (g/L)	124 (109-134)	122 (106-133)	0.706
Ddimer (ug/ml)	1.341 (0.842-2.94)	1.21 (0.844-2.548)	0.193
PCT (ng/ml)	0.1 (0.05-0.255)	0.1 (0.06-0.23)	0.795
CRP (mg/L)	12.66 (5.125-33.065)	11.09 (4.8-25.79)	0.142
Variable	Training (*n*=463)	Testing (*n*=196)	*P*
Gender			0.336
Male	304 (65.66)	57 (69.5)	
Female	159 (34.34)	25 (30.5)	
Age	72 (67-80)	75 (68-81)	0.052
BMI	23 (21.22-25.1)	23 (22.58-24.91)	0.101
Operation history	224 (48.38)	95 (48.47)	0.983
BTH	29 (6.26)	7 (3.57)	0.164
COPD	35 (7.56)	17 (8.67)	0.630
Diabetes	114 (24.62)	40 (20.41)	0.240
Hypertension	221 (47.73)	95 (48.47)	0.860
CHD	103 (22.25)	35 (17.86)	0.210
AID	18 (3.89)	3 (1.53)	0.120
Cough	420 (90.71)	171 (87.24)	0.180
Fever	389 (84.02)	175 (89.29)	0.080
Diagnostic classification			0.406
Common type	283 (61.12)	113 (57.65)	
Heavy type	180 (38.88)	83 (42.35)	
Time			0.861
≤7 Days	162 (34.99)	71 (36.22)	
(7–14 ] Days	171 (36.93)	68 (34.69)	
>14 Days	130 (28.08)	57 (29.08)	
WBC (×10^9^)	6.19 (4.66-8.57)	5.86 (4.15-8.22)	0.060
NEUT (×10^9^)	4.67 (3.15-7.14)	4.335 (2.905-6.33)	0.099
LYM (×10^9^)	0.82 (0.51-1.175)	0.82 (0.55-1.15)	0.787
EOS (×10^9^)	0.02 (0-0.075)	0.02 (0-0.06)	0.342
LYMs%	13.9 (7.25-21.2)	15.5 (8-22.25)	0.086
NEUT%	76.3 (67.5-85.35)	76.5 (66-86.75))	0.862
RBC (×10^12^)	4.01 (3.53-4.38)	4.19 (3.89-4.665)	<0.001
Hb (g/L)	124 (109-134)	130.5 (119-144.5)	<0.001
Ddimer (ug/ml)	1.341 (0.842-2.94)	1.345 (0.665-3.13)	0.119
PCT (ng/ml)	0.1 (0.05-0.255)	0.0985 (0.0515-0.255)	0.720
CRP (mg/L)	12.66 (5.125-33.065)	11.475 (3.915-33.66)	0.389

NEUT stands for neutrophil count, NEUT% for neutrophil percentage, LYM for lymphocyte count, LYM% for lymphocyte percentage, EOS for eosinophil count, Hb for hemoglobin, BTH for blood transfusion history, COPD for chronic obstructive pulmonary disease, CHD for coronary heart disease, and AID for autoimmune disease.

### Nomogram development

**Figure 3:**
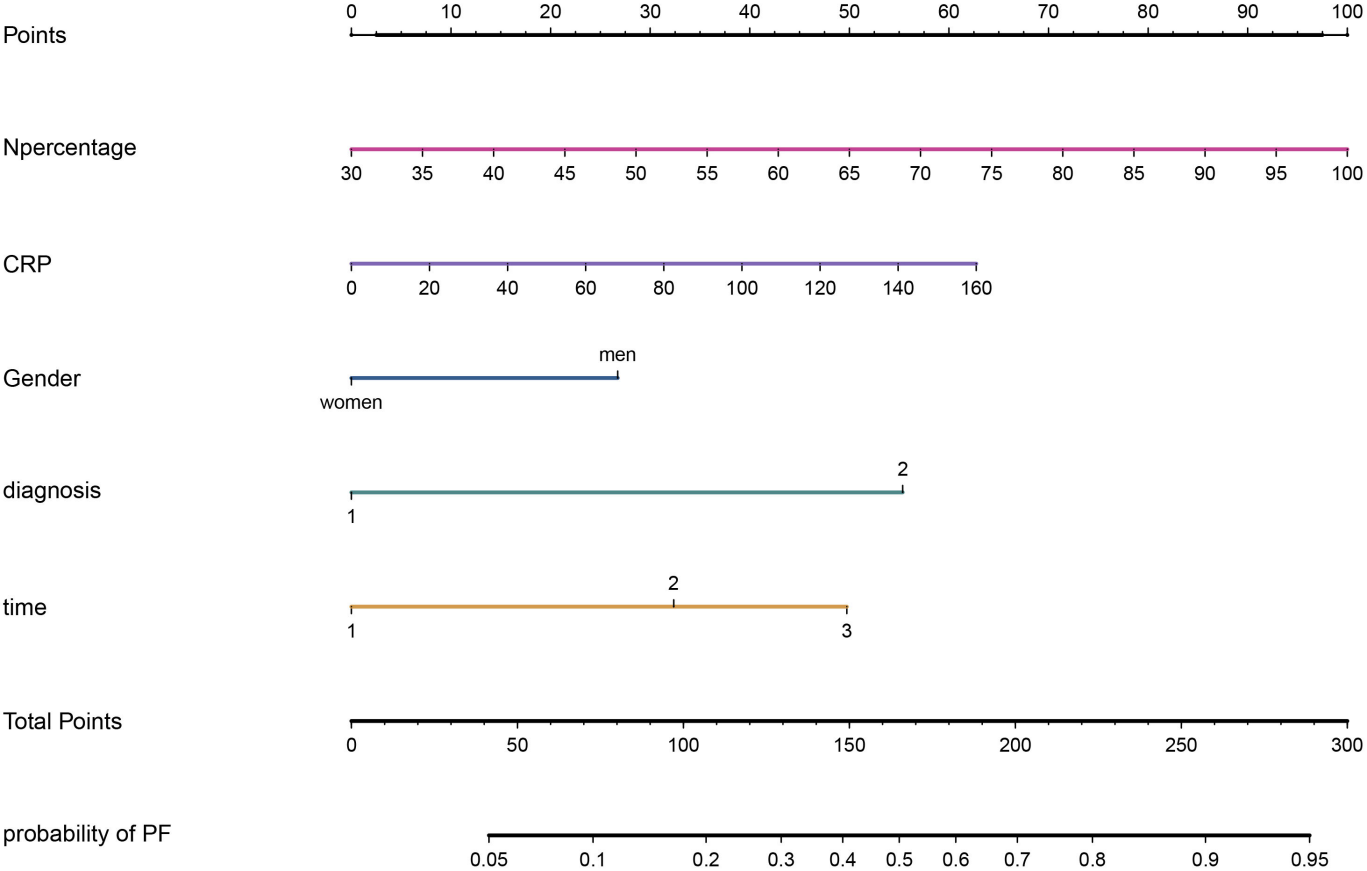
A nomogram for PF in elderly patients after SARS-CoV-2 infection.

Multivariate logistic regression analysis confirmed that the percentage of neutrophils, CRP, gender, diagnostic classification, and the time from symptom onset to hospitalization were independent predictors for PF in elderly patients with SARS-CoV-2 infection. A nomogram was developed using the regression coefficients (0.034, 0.009, 0.637, 1.319, and 1.185) of these predictors, implemented *via* the “rms” package in R software (**Figure 3**). In the nomogram, the length of the lines corresponds to the weight of each predictor, with the percentage of neutrophils having the highest weight, followed by CRP and diagnostic classification. Gender has the least impact. The percentage of neutrophils was assigned 100 points, and the remaining four predictors were assigned 62.72, 26.74, 55.35, and 49.74 points, respectively, based on the ratio of regression coefficients. The total score was calculated by summing the points for all five predictors, with the corresponding risk of PF determined by the vertical line corresponding to the total score.

### Multiple validations of the Nomogram

**Figure 4:**
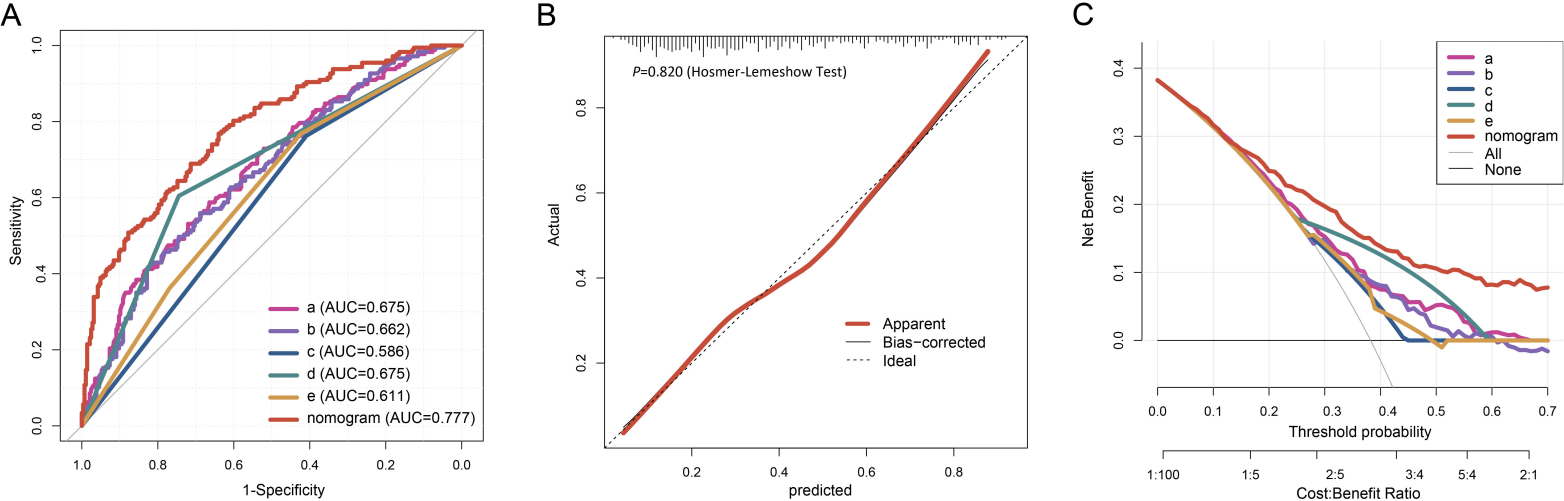
Evaluation of the nomogram for PF of the elderly patients infected with SARS-COV-2. The ROC curve **(A)**, calibration plot **(B)**, and DCA **(C)** of the nomogram in the training cohort. Calibration plot of the nomogram. The gray dashed line represents ideal calibration. The red and black solid lines indicate the original model (prone to overfitting) and Bootstrap-corrected model, respectively. In the DCA graph, "All" refers to the assumption that all patients develop PF, and "None" refers to the assumption that no patients develop PF. 5 key predictors are listed in the annotation: a. Percentage of neutrophils; b. CRP; c. Gender; d. Diagnostic classification; e. Time from symptom onset to hospital admission. AUC represents the area under the curve.

In the internal validation using the training cohort, the nomogram accurately predicted the likelihood of developing PF after SARS-CoV-2 infection, with an AUC of 0.777 (95% CI, 0.734 - 0.820; **Figure 4A**). The Hosmer-Lemeshow test yielded *χ^2^* = 4.393, *P* = 0.820, and the calibration curve demonstrated good agreement between the observed and predicted probabilities (**Figure 4B**). DCA revealed that the nomogram’s net benefit significantly exceeded that of each independent predictor (**Figure 4C**).

**Figure 5:**
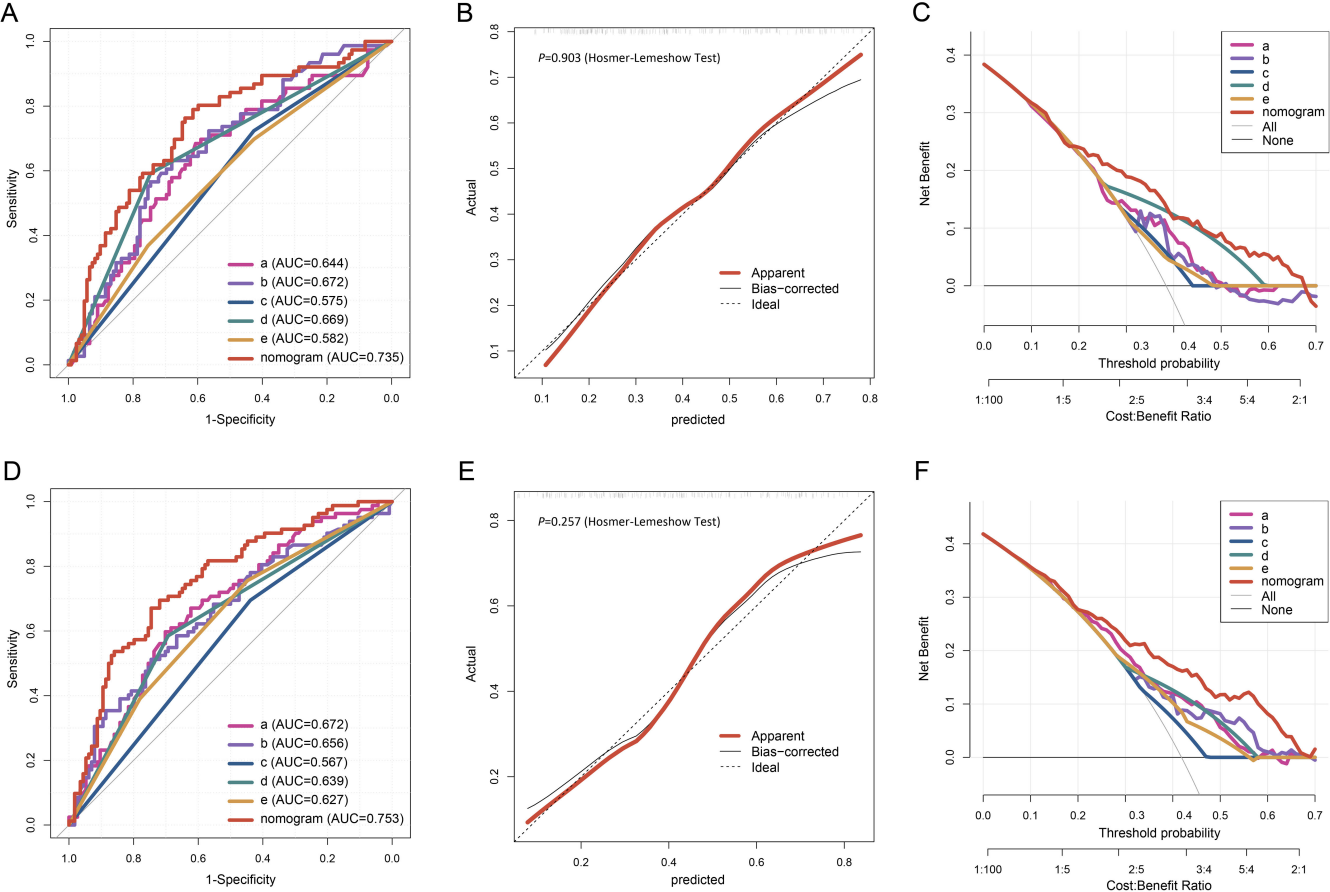
The ROC curve **(A)**, calibration plot **(B)**, and DCA **(C)** of the nomogram in the validation cohort. The ROC curve **(D)**, calibration plot **(E)**, and DCA **(F)** of the nomogram in the testing cohort.

In the external validation, the nomogram’s AUC for 198 elderly patients was 0.735 (95% CI, 0.663 - 0.807) (**Figure 5A**). The Hosmer-Lemeshow test result was *χ^2^* = 3.456, *P* = 0.903 (**Figure 5B**). DCA confirmed that the nomogram provided superior clinical prediction performance compared to individual predictors (**Figure 5C**). To further evaluate the model’s validity and generalizability, a test cohort was established with 196 patients from a different hospital. In this cohort, the AUC was 0.753 (95% CI, 0.685 - 0.821; **Figure 5D**), and the Hosmer-Lemeshow test result was *χ^2^* = 10.123, *P* = 0.257 (**Figure 5E**), indicating good accuracy. Additionally, the calibration curve demonstrated high predictive consistency. DCA further suggested that the prediction model showed similar efficacy across the training, validation, and test cohorts (**Figure 5F**).

### Determination of the optimal cut-off value for the nomogram

Using data from all 857 patients in this study, we calculated the sensitivity, specificity, positive predictive value (PPV), negative predictive value (NPV), positive likelihood ratio (LR+), negative likelihood ratio (LR-), positive utility index (UI+), negative utility index (UI-), diagnostic odds ratio (DOR), and the Youden index at various cut-off points of the nomogram (**Table 2**). Furthermore, based on the principle of optimizing the balance between sensitivity and specificity, the optimal cut-off value of the nomogram was determined to be 131.026 (sensitivity 0.773, specificity 0.621). Elderly patients with a nomogram score ≥ 131.026 were classified as high-risk for PF, and early targeted management and intervention were recommended for these individuals.

**Table 2:** A column chart and corresponding scores for evaluating performance parameters at different critical points.

Cutoff points	Sensitivity	Specificity	PPV	NPV	LR+	LR-	DOR	UI+	UI-	Youden index
71.523	0.979	0.142	0.423	0.914	1.141	0.147	7.740	0.414	0.130	0.121
85.563	0.937	0.228	0.438	0.850	1.214	0.275	4.415	0.410	0.194	0.165
99.887	0.919	0.343	0.473	0.869	1.399	0.235	5.953	0.435	0.298	0.262
110.358	0.878	0.441	0.502	0.849	1.569	0.278	5.648	0.440	0.374	0.318
118.053	0.833	0.504	0.519	0.824	1.679	0.332	5.059	0.432	0.415	0.337
126.783	0.806	0.584	0.554	0.824	1.939	0.332	5.838	0.447	0.482	0.390
131.026	0.773	0.621	0.567	0.810	2.038	0.366	5.577	0.438	0.503	0.394
133.045	0.761	0.630	0.569	0.804	2.059	0.379	5.434	0.433	0.507	0.391
138.157	0.716	0.661	0.576	0.784	2.113	0.429	4.924	0.412	0.518	0.377
145.028	0.666	0.713	0.598	0.769	2.317	0.469	4.938	0.398	0.548	0.378
154.221	0.594	0.782	0.636	0.750	2.720	0.519	5.237	0.378	0.586	0.376
162.658	0.533	0.822	0.657	0.734	2.997	0.568	5.276	0.350	0.603	0.355
176.385	0.460	0.881	0.713	0.718	3.870	0.613	6.313	0.328	0.632	0.341
188.851	0.383	0.925	0.766	0.701	5.139	0.666	7.711	0.294	0.649	0.309
210.402	0.191	0.966	0.780	0.650	5.540	0.838	6.613	0.149	0.628	0.157

PPV, positive predictive value; NPV, negative predictive value; LR+, positive likelihood ratio; LR−, negative likelihood ratio; UI+, positive utility index; UI-, negative utility index; DOR, diagnostic odds ratio.

## Discussion

Following SARS-CoV-2 infection, most patients exhibit changes in lung imaging (Xu et al., 2020; Wendisch et al., 2021). According to the Fleischner Society criteria, abnormal imaging findings in the residual lung can be categorized into fibrosis and non-fibrosis (Bocchino et al., 2023). The diagnosis of PF due to SARS-CoV-2 primarily depends on chest HRCT characteristics (Li et al., 2021). Typical manifestations include traction bronchiectasis and irregular interfaces (such as streak shadows, reticular patterns, honeycombing, or structural deformation), accompanied by symptoms like progressively worsening dyspnea and decreased oxygenation (Huang et al., 2021; Solomon et al., 2021). Pathologically, PF is characterized by re-epithelialization, fibroblast activation, and increased collagen deposition (Wijsenbeek et al., 2022). SARS-CoV-2 infection accelerates the development of PF through direct lung tissue damage from viral invasion, dysregulated immune responses, abnormal extracellular matrix deposition, and tissue remodeling, mechanisms similar to those seen with other respiratory viruses. This process involves the activation of various molecules and signaling pathways, including TGF-β, TNF-α, IL-1β, IL-6, IL-13, and autophagy inhibition (Huang and Tang, 2021; Bailey et al., 2024). Once PF develops, patients often have a poor prognosis and require prompt antiviral and anti-fibrotic treatments (Soni et al., 2024). The elderly are particularly vulnerable to SARS-CoV-2 (Liu et al., 2020), with a higher likelihood of developing severe pneumonia and PF, resulting in worse overall outcomes (Al-Salameh et al., 2021; Farshbafnadi et al., 2021). Therefore, early identification of elderly individuals at high risk for PF, along with the development and implementation of personalized treatment plans, is crucial for improving the prognosis of the elderly population infected with SARS-CoV-2.

This study established a risk prediction model incorporating five risk factors for PF in elderly patients with SARS-CoV-2 infection. The total prediction score is calculated by summing the scores of these five independent variables, with the resulting score used to estimate the probability of PF. Notably, the AUC and DCA demonstrated the high accuracy of this prediction model. Among the five predictors, male gender was identified as a significant risk factor for PF after COVID-19 infection, both in younger and older patients (Ley et al., 2012; Kam et al., 2019; Johnston et al., 2023). The underlying mechanism is likely related to the effect of androgens in exacerbating fibrotic progression through enhanced inflammatory responses and fibroblast activation (Becerra-Diaz et al., 2020).

As a key inflammatory mediator, the dynamic changes in serum CRP levels not only accurately reflect systemic inflammation intensity (Zhang et al., 2023) but also exert multiple pro-fibrotic effects during PF. Following SARS-CoV-2 infection, CRP activates the classical complement pathway by binding to complement component C1q, while also interacting with Fcγ receptors on macrophage surfaces through its ligand-binding domain. These mechanisms synergistically enhance monocyte-macrophage chemotaxis and infiltration into lung tissue, promote the release of inflammatory cytokines, and ultimately establish a pro-fibrotic inflammatory microenvironment (Junqueira et al., 2022; Lage et al., 2022; Meroni et al., 2023). CRP demonstrates a significant correlation with mortality prognosis in SARS-CoV-2-infected patients, with its elevation magnitude positively associated with both the risk of pulmonary fibrotic lesion development and the degree of radiographic progression (Xie et al., 2022; Ouyang et al., 2024). Additionally, CRP has shown strong predictive performance in forecasting the occurrence of rheumatoid arthritis-associated PF (Xue et al., 2022). Elevated CRP levels have been independently linked to reduced 5-year survival rates in patients with PF (Stock et al., 2024). In the progression of post-SARS-CoV-2 infection PF, this study revealed a significantly increased risk of fibrotic development in elderly infected individuals with higher CRP levels. Notably, CRP was identified as a key factor associated with the occurrence of SARS-CoV-2-induced PF in elderly patients.

Neutrophils, macrophages, and dendritic cells are crucial lung cell populations and act as first responders during lung infection or injury (Chen et al., 2021; Graf et al., 2023). Neutrophils play a dual regulatory role in the development of PF following SARS-CoV-2 infection. As primary effector cells of innate immunity, neutrophils exhibit abnormal activation during infection. By releasing neutrophil extracellular traps (NETs), they contribute to a thromboinflammatory microenvironment. This process involves platelet activation and initiation of the coagulation cascade (Lee et al., 2021; Herro and Grimes, 2024), as well as direct participation in pathological tissue remodeling during lung injury repair *via* NET-mediated cascades (Ackermann et al., 2021). On the pro-inflammatory level, effector molecules such as cathepsins released from NETs degrade components of the alveolar epithelial basement membrane, compromising the integrity of the alveolar-capillary barrier (Pulavendran et al., 2020). Simultaneously, NETs activate the NF-κB signaling pathway in lung interstitial fibroblasts through TLR-9 receptors, inducing excessive secretion of pro-fibrotic factors like IL-6 (Shao et al., 2022). These mechanisms ultimately drive abnormal fibroblast proliferation and excessive extracellular matrix deposition, leading to irreversible fibrotic lesions (Negreros and Flores-Suárez, 2021). Patients with PF exhibit significantly higher neutrophil ratios compared to non-fibrotic controls (Lang et al., 2021). Notably, a neutrophil ratio exceeding 68.3% has been identified as an independent risk factor for predicting poor prognosis in patients with idiopathic PF (Cheng et al., 2023). This study also confirmed that elderly patients with PF have significantly higher neutrophil proportions compared to those without fibrosis.

The time from symptom onset to hospitalization is another important, yet often overlooked, factor that significantly predicts SARS-CoV-2-induced PF in the elderly. Among critically ill patients, lung parenchymal damage tends to be more severe, and delayed hospital admission may contribute to prolonged viral replication and uncontrolled inflammatory responses (Smulowitz et al., 2021). Delayed admission has been identified as a significant predictor of increased mortality rates in severe cases (Akhtar et al., 2021).

This study highlights that combining clinical features with laboratory indicators enhances clinicians’ ability to assess the risk of PF in elderly SARS-CoV-2-infected patients. Through multivariate logistic regression analysis, key factors contributing to the development of PF were identified, including percentage of neutrophils, CRP, gender, diagnostic classification, and the time from symptom onset to admission. These factors are commonly available and easily accessible. Moreover, the prediction model developed in this study simplifies complex clinical data into an intuitive nomogram, which was validated using ROC curve, calibration curve, and DCA, ultimately demonstrating good predictive efficacy. Most existing prediction models for PF focus on the general population, often neglecting the unique characteristics of the elderly (Lee et al., 2022; Ouyang et al., 2024; Xu et al., 2024). While previous studies have identified potential risk factors, such as comorbidities, obesity, and abnormal laboratory indicators (D’Agnillo et al., 2021; Schuliga et al., 2021), comprehensive validation of their predictive value remains insufficient. In contrast, this study specifically targets the elderly population, addressing a critical gap in population-specific prediction models. By innovatively incorporating the temporal dimension (time from symptom onset to hospital admission) along with laboratory indicators in the nomogram, this approach allows for real-time clinical assessment. This approach facilitates early intervention, such as initiating antifibrotic therapies (Pirfenidone/Nintedanib) in combination with pulmonary rehabilitation for high-risk patients. The model shows consistent stability through both validation and external testing phases, underscoring its robust clinical generalizability. Clinicians can thus use this model to more effectively and efficiently predict the risk of PF in elderly patients infected with SARS-CoV-2.

This study has several limitations. First, it is a retrospective study, with data primarily collected from electronic records, which are inherently limited. Additionally, as the data were sourced from two different hospitals within the similar healthcare system, the generalizability of the findings to broader populations requires further validation in future studies. Second, some patients may have received pre-admission interventions, such as traditional Chinese medicine therapies, antiviral treatments, or glucocorticoids. These treatments could influence the intensity of inflammatory responses and tissue repair processes, potentially affecting the development of PF. Furthermore, such interventions might have impacted post-admission laboratory parameters, including serum inflammatory markers and chest HRCT imaging findings. Despite these limitations, internal and external validations were performed using data from the first hospital, along with supplementary data from the second hospital (collected during the same period), to test the prediction model, thereby ensuring its accuracy and applicability.

## Conclusion

This study developed a nomogram for predicting secondary PF in the elderly following SARS-CoV-2 infection. Rigorous internal and external validations demonstrated that the model exhibits excellent discrimination and calibration, making it a useful, intuitive, and individualized clinical tool for assessing the risk of PF in elderly SARS-CoV-2-infected patients. Ultimately, it can assist clinicians in early management and intervention.

## Data Availability

The datasets supporting the findings of this study are available from the corresponding author upon reasonable request.
