# Supplementary material for: Retrospective cohort analysis on predicting pulmonary fibrosis in elderly SARS-CoV-2-infected patients
Source: Front Cell Infect Microbiol. 2025 Jun 6;15:1587321. doi: 10.3389/fcimb.2025.1587321 (PMC12179164; doi:10.3389/fcimb.2025.1587321)

Supplementary Figure 1. Typical HRCT images of pulmonary fibrosis and good pulmonary shadow absorption in patients infected with SARS-CoV-2. Figure A shows the gradual worsening of pulmonary shadows evolving into pulmonary fibrosis, with honeycomb-like changes. Figure B shows the gradual absorption of pulmonary shadows, indicating improvement.


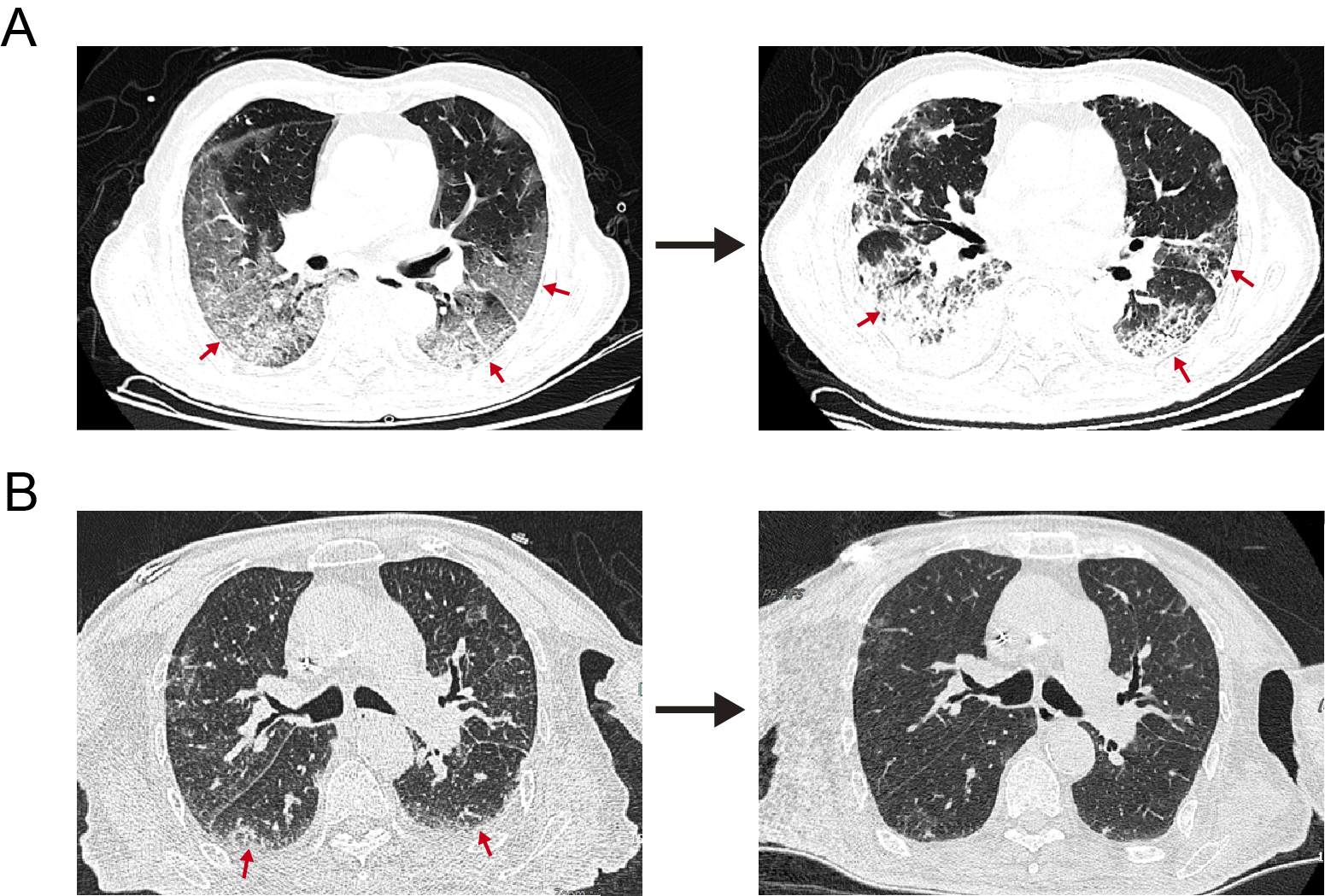

Supplement: Supplementary file 1 [file Table1.docx]
